# Multi-Modal Deep Learning for Weeds Detection in Wheat Field Based on RGB-D Images

**DOI:** 10.3389/fpls.2021.732968

**Published:** 2021-11-05

**Authors:** Ke Xu, Yan Zhu, Weixing Cao, Xiaoping Jiang, Zhijian Jiang, Shuailong Li, Jun Ni

**Affiliations:** ^1^College of Agriculture, Nanjing Agricultural University, Nanjing, China; ^2^National Engineering and Technology Center for Information Agriculture, Nanjing, China; ^3^Engineering Research Center of Smart Agriculture, Ministry of Education, Nanjing, China; ^4^Jiangsu Key Laboratory for Information Agriculture, Nanjing, China; ^5^Jiangsu Collaborative Innovation Center for the Technology and Application of Internet of Things, Nanjing, China; ^6^College of Artificial Intelligence, Nanjing Agricultural University, Nanjing, China

**Keywords:** weeds detection, RGB-D image, multi-modal deep learning, machine learning, three-channel network

## Abstract

Single-modal images carry limited information for features representation, and RGB images fail to detect grass weeds in wheat fields because of their similarity to wheat in shape. We propose a framework based on multi-modal information fusion for accurate detection of weeds in wheat fields in a natural environment, overcoming the limitation of single modality in weeds detection. Firstly, we recode the single-channel depth image into a new three-channel image like the structure of RGB image, which is suitable for feature extraction of convolutional neural network (CNN). Secondly, the multi-scale object detection is realized by fusing the feature maps output by different convolutional layers. The three-channel network structure is designed to take into account the independence of RGB and depth information, respectively, and the complementarity of multi-modal information, and the integrated learning is carried out by weight allocation at the decision level to realize the effective fusion of multi-modal information. The experimental results show that compared with the weed detection method based on RGB image, the accuracy of our method is significantly improved. Experiments with integrated learning shows that mean average precision (*mAP*) of 36.1% for grass weeds and 42.9% for broad-leaf weeds, and the overall detection precision, as indicated by intersection over ground truth (*IoG*), is 89.3%, with weights of RGB and depth images at α = 0.4 and β = 0.3. The results suggest that our methods can accurately detect the dominant species of weeds in wheat fields, and that multi-modal fusion can effectively improve object detection performance.

## Introduction

Weeds are a major biological problem that limits the yield and quality of wheat by competing for light, water, fertilizer, and space (Munier-Jolain et al., 2013; Fahad et al., 2015). There are both grass and broad-leaf weeds (Gaba et al., 2010). Grass weeds have narrow, long leaves very similar to those of wheat, and consist mainly of *Echinochloa crusgalli*, *Avena fatua*, and *Aegilops tauschii*. Broad-leaf weeds look different, and include *Pharbitis nil*, *Galium spurium*, *Veronica didyma*, *Capsella bursa-pastoris*, and *Convolvulus arvensis*. Changes in farming practice and the introduction of new wheat varieties have led to significant changes in the species and occurrence of weeds. Grass weeds have invaded and dominated wheat fields, and like broad-leaf weeds, they threaten production (Ulber et al., 2009). They diminish wheat grain filling and have a greater impact on growth and yield (Siddiqui et al., 2010). Grass weeds have morphological characteristics and living habits similar to those of wheat, which interfere with their recognition.

Chemical herbicides have become the primary means of farmland weeds management worldwide because of their high efficiency (Jaime and Ricardo, 2017; Kniss, 2017). Due to the lack of information on weeds species and distribution, they are sprayed over large areas, resulting in overuse, low utilization, and serious pollution. Although herbicides can directly kill object wild plants, excessive use will cause serious environmental pollution (Rose et al., 2016), decrease the yield and quality of agricultural products, and reduce the efficiency of agricultural production. Site-specific weeds management (SSWM) is an important solution to herbicide overuse, whose study includes the aspects of crop and weeds detection systems, decision-making algorithms for herbicide application, and weeds control implementation (Camille et al., 2008; Christensen et al., 2010), among which the recognition and localization of weeds in fields are key issues.

Since images with high spatial resolution are usually easily available and not costly, they are favored in the integration and application of precision weeds management and adjustable spraying systems. Hence, weeds detection based on digital images is a key technical tool for the accurate recognition and localization of weeds in farmlands (Bakhshipour et al., 2017). Weeds detection with wheat field images using traditional machine learning methods usually requires the selection of an object area with a sliding window. Manually designed features, such as the color, location, morphology, and texture of wheat and weeds, are analyzed and extracted from wheat field images (Tellaeche et al., 2008; Petra et al., 2018). This process fails in multi-scale object detection tasks, the time complexity is high, and manually designed features are sensitive to sample variation (Pflanz et al., 2018; Xu et al., 2020a). Deep learning approaches based on CNNs avoid the use of manually designed features, can well detect objects of different sizes, and have been used in the study of weeds detection to significantly improve recognition efficiency (Patrícioa and Riederb, 2018; Smith et al., 2019; Alsamhi et al., 2021; Saleh et al., 2021). However, most weeds detection algorithms are based on the input of single-modal images (RGB), and the limited information makes it difficult to recognize different weeds species (Alessandro et al., 2017; Bah et al., 2018; Huang et al., 2018b). In particular, weeds detection in wheat fields is seriously restricted by the similar shapes of grass weeds and wheat, and the lack of recognizability in RGB images.

Studies have demonstrated that the fusion of multi-modal data can effectively improve the robustness of object detection in unfavorable environments, and the combination of RGB, depth and infrared information provides more richer feature space, which facilitates precise classification and detection (Haque et al., 2020). Since different modalities represent the same scene in different ways, their independence and complementarity can be used to improve the precision of object detection. RGB images, which contain color and texture information, and depth images, which contain geometric structure information, are widely used in object detection tasks because of their high complementarity (Qi et al., 2018). The pixel value in a depth image reflects the distance between the object and the sensor, and effectively describes the geometric characteristics of the object surface in an image. A depth image is therefore an effective supplement to an RGB image. Plant height is an important feature of growth status, which differs greatly between crops and weeds due to growth competition (Piron et al., 2009; Zhang and Grift, 2012). Therefore, we fuse the multi-modal information from RGB and depth images of wheat and weeds to detect weeds in wheat fields. Our work can be summarized as follows:

(1) A weeds-in-wheat-field RGB-D dataset for object detection is proposed, including 1,228 RGB images and corresponding depth images, and the weed areas are labeled as broad-leaf and grass.
(2) To address the CNN’s inability to extract abundant features from single-channel depth images, they are recoded by simulating the RGB image structure to generate new structure images that contain more geometric information and are more suitable for CNN-based feature learning.
(3) According to the concept of multiscale object detection and considering the independence and complementarity of multi-modal data, a three-channel network for weeds detection is proposed from the perspectives of feature-level fusion and decision-level fusion.

## Materials and Methods

### Experimental Design

**FIGURE 1:**
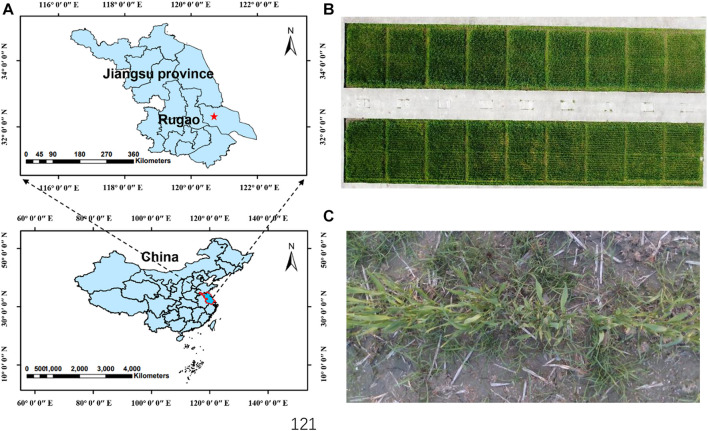
**(A)** Experimental site; **(B)** Images of all plots; **(C)** RGB image of wheat field. The red star represents the location of our experiments in the map.

Experiments on wheat and weeds were carried out from December 2017 to April 2020 at the demonstration base of the National Engineering and Technology Center for Information Agriculture in Rugao County, Nantong City, Jiangsu Province, China. The experimental area was 50 m long and 12 m wide (Figure 1). Weeds were not controlled during field management, and seeds of six weeds species commonly associated with wheat were randomly sown to simulate weeds growth in the open field. *Alopecurus aequalis*, *Poa annua*, *Bromus japonicus*, and *E*. *crusgalli* are grass weeds; *Amaranthus retroflexus* and *C*. *bursa-pastoris* are broad-leaf weeds; and the species composition was similar to that of actual weeds species in wheat fields.

### Image Acquisition and Preprocessing

**FIGURE 2:**
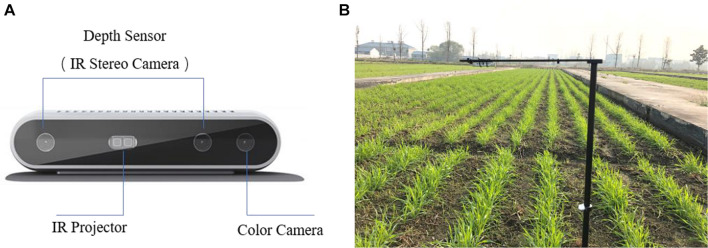
**(A)** Intel RealSense D415; **(B)** Equipment setup for field image acquisition.

Wheat field images were acquired using an Intel RealSense Depth Camera D415 (99 mm × 20 mm × 23 mm), an RGB-D camera that adopts active infrared stereo vision technology. As shown in Figure 2A, there were two infrared stereo cameras, an infrared projector, and a color sensor. The infrared stereo cameras generate depth images, and the color sensor generates RGB images, both with a resolution of 1,280 × 720. RGB and depth field images under natural conditions were obtained at wheat tillering and jointing stages. Image collection was carried out under clear and windless weather conditions. The camera was 70 cm above the crop canopy, and set up in the field as shown in Figure 2B. Images were transmitted to a computer in real time via USB 3.0.

Since the RGB and depth images had different origins, there is a mismatch problem between the data of different modalities, that is, the same object has a certain degree of position deviation on the images of different modalities. In order to share unified labeling results between RGB images and depth images in subsequent image samples and reduce the impact of mismatch on subsequent feature fusion, coordinate transformation is used first to align depth images and RGB images. The process of image alignment is firstly to restore the depth point of the depth image coordinate system to the world coordinate system, and then to convert the depth point of the world coordinate system to the RGB image coordinate system. The internal parameters matrix, rotation matrix, and translation vector of the depth camera and RGB camera were obtained from the System Design Kit (SDK) provided by Intel. Data loss (void) occurred in depth images due to lighting conditions, infrared reflective properties of object surface materials, and shielding, and depth images were repaired using a hole filling (HF) algorithm (Xu et al., 2020b) to obtain complete depth information for subsequent feature extraction.

### Recoding Depth Images

A depth image contains data captured by a depth camera that reflect the distance between the object and the camera. A depth image provides information on object shape and geometry that are lost in RGB images but crucial for object detection. In many object detection studies based on multi-modal information, depth images provide supplementary information to RGB images and improve the performance of object detection (Gupta et al., 2010; Hedau et al., 2010). However, the original depth information is less representative. In particular, feature extraction from depth images with a CNN generates feature maps of distance rather than geometric structures with physical significance. Therefore, single-channel depth images were transformed to three-channel images by recoding the original images to make them more representative and structurally similar to RGB images. The three channels of recoded images are phase, height above ground, and angle with gravity, and recoded images are referred to as PHA images. Phase was calculated according to the mechanism by which depth information is generated (Cai et al., 2017),


(1) d=n⋅2⁢π⁢l+ϕ⁢l

where *n* ∈ *ℕ*, *l* ∈ *ℕ*, and 2π*l* is the uniqueness range of the camera. The natural number *n* ensures ϕ ∈ [0,2π]. The maximum distance in the depth image *d*_*max*_ was identified, and the relative distance between object and sensor was converted to height above ground,


(2) $$H=d_{\max}-d\,\left(i,j\right)$$

where *d*(*i*,*j*) is the depth in image coordinates (*i*,*j*). The third channel (angle with gravity) is the angle between the local surface of a pixel and the direction of gravity (Gupta et al., 2013),


(3) $$\min_{g:{||g||}_{2}=1}\sum_{n_{i}\in N_{1}}\cos^{2}\left(\theta \,\left(n_{i},g\right)\right)+\sum_{n_{j}\in N_{2}}\sin^{2}\left(\theta \,\left(n_{j},g\right)\right),$$

Where *g* is the direction of gravity, *N*_*1*_ represents the set of normals parallel to the direction of gravity, *N*_*2*_ represents the set of normals perpendicular to the direction of gravity, *n*_*1*_ and *n*_*2*_ represent some element in *N*_*1*_ and *N*_*2*_, respectively. And θ is the Angle between two vectors.

### Weeds Object Detection Network Based on Multi-Modal Information

There are two primary approaches to detect objects based on multi-modal information from RGB-D: (1) to use a depth image as an additional channel of the RGB image (Couprie et al., 2014); and (2) to separately learn features from RGB and depth images (Wang et al., 2015). However, these methods can neither extract fine geometric features from depth images nor make full use of the complementarity of different modalities. We designed a network by considering the common features (complementarity) between the two modalities (RGB and depth) and the unique features (independence) learned from single modalities.

**FIGURE 3:**
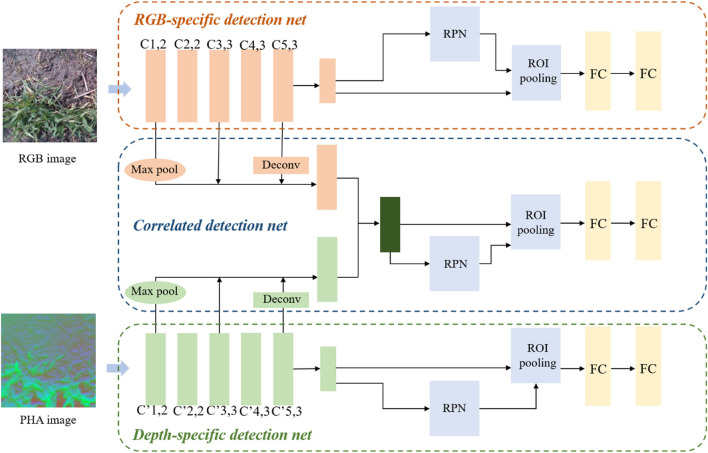
Weeds detection network architecture.


The network architecture is shown in Figure 3. We designed a three-channel CNN to learn different features from multi-modal information, including two channels for learning RGB- and depth-specific features, and one for learning RGB-D-correlated features. Each network was designed based on Faster R-CNN with VGG16 as a backbone. Since the object area of weeds varied greatly and some were thin and small, we introduced multiscale object detection when designing the correlated detection net. The concept of multiscale representation in CNNs has been demonstrated in previous studies. Low-level feature maps have smaller receptive fields and larger scales, and they contain less semantic information and more low-level feature information such as edges and colors. High-level feature maps contain more semantic information and high-level feature information such as object parts and components (Bell et al., 2016; Kong et al., 2016). Therefore, object detection using the last feature map is not favorable for the detection of multiscale and small objects. Therefore, when designing the architecture of correlated detection net, the basic idea is to utilize the advantages of different receptive fields in detecting targets of different scales so that the network can better deal with multi-scale targets and improve the overall detection accuracy. We used the structure of a hypernet as a reference in designing the structure of a correlated detection net (Kong et al., 2016), fusing the feature maps after the first, third, and fifth convolutional blocks in RGB- and depth-specific detection nets. Because layers had different feature map dimensions, max pool was used in the first layer, and deconv in the fifth layer, to facilitate calculation. To enhance the learning of complementary features by the correlated detection net, instead of directly connecting via add and concat, a previously described method (Xu et al., 2017) was used in the ultimate fusion of feature maps, and the fused feature map was defined as:

(4) $$f_{corr}=\sqrt{f_{RGB}\circ \;f_{Depth}}$$

where *f*_*RGB*_ and *f*_*Depth*_ denote the feature maps generated by CNN in RGB- and depth-specific detection nets, respectively, and ° denotes the Hadamard product.

In order to generate multi-modal object proposals, three Region Proposal Networks (RPNs; Ren et al., 2017) are slid over last feature maps. One is for modality-correlated object estimation and the other two are for modality-specific object estimation. The loss function of RPN network is divided into two parts: the boundary-box regression loss function and the classification loss function. For bounding box regression, given an anchor box with (*x*_*a*_,*y*_*a*_,*w*_*a*_,*h*_*a*_), bounding box regression is developed to predict deviations $t^{*}=\left(t_{x}^{*},t_{y}^{*},t_{w}^{*},t_{h}^{*}\right)$ following (Felzenszwalb et al., 2010; Girshick et al., 2013):


(5) $$\left\{ \begin{array}{c} t_{x}^{*}=\left(x^{*}-x_{a}\right)/w_{a}\\ t_{y}^{*}=\left(y^{*}-y_{a}\right)/h_{a},\\ t_{w}^{*}=\log\left(w^{*}/w_{a}\right)\\ t_{h}^{*}=\log\left(h^{*}/h_{a}\right) \end{array}\right.$$

Where *x*,*y*,*w* and *h* denote the bounding box’s center coordinates and its width and height. *x*^∗^,*y*^∗^,*w*^∗^,*h*^∗^ and *x*_*a*_,*y*_*a*_,*w*_*a*_,*h*_*a*_ are for the ground-truth box and anchor box, respectively. *Smooth L*1 (Girshick, 2015) is adopted to calculate the bounding box regression loss.


(6) $$S\,m\,o\,o\,t\,h\,\,L\,1=\left\{ \begin{array}{c} 0.5\,x^{2}\;i\,f\,\left|x\right|<1,\\ \left|x-0.5\right|\;o\,t\,h\,e\,r\,w\,i\,s\,e \end{array}\right.$$

With these definitions, the object estimation multi-task loss *L* is defined as:


(7) $$L\,\left(\left\{p_{i}\right\},\left\{t_{i}\right\}\right)=\frac{1}{N_{c\,l\,s}}\,\sum_{i}L_{c\,l\,s}\,\left(p_{i},p_{i}^{*}\right)+\lambda \,\frac{1}{N_{r\,e\,g}}\,\sum_{i}p_{i}^{*}\,S\,m\,o\,o\,t\,h\,\,L\,1\,\left(t_{i}-t_{i}^{*}\right)$$

where the mini-batch size is ignored. *i* represents the index of an anchor point, *p*_*i*_ and $p_{i}^{*}$ are the predicted object probability of an anchor and ground-truth label. If the anchor is positive, $p_{i}^{*}=1$. And if the anchor is negative, $p_{i}^{*}=0$. Two types of anchors are treated as positive: the anchors with the highest intersection over union (*IoU*) overlap with a ground-truth box, and the ones that have an *IoU* overlap higher than 0.7 with any ground-truth box. *L*_*cls*_ is log loss over. The two terms in Eq. (7) are normalized by *N*_*cls*_ and *N*_*reg*_ and weighted by a balancing parameter λ. The former is normalized by the mini-batch size and the latter is normalized by the number of anchor locations. The modality-correlated RPN and modality-specific RPNs are trained simultaneously with the same supervision.

To better exploit the complementarity of multi-modal data, we fused data at the decision level of the algorithm for ensemble learning and assigned weights to detection results of the three-channel network. The equation is as follows:


(8) $$G\,\left(x\right)=\alpha \,g_{R\,G\,B}\,\left(x\right)+\beta \,g_{P\,H\,A}\,\left(x\right)+\left(1-\alpha -\beta \right)\,g_{c\,o\,r\,r}\,\left(x\right)$$

where, *g*(⋅) is the output of detection network, α, β (α≥0,β≥0,α + β≤1) are the ensemble weights for RGB and depth branch, respectively. α, β vary in [0,1] with a step size of 0.05.

### Datasets and Model Training Methods

To increase the robustness of the object detection network, we performed image data enhancement by rotation and flipping. The resulting multi-modal weeds in the wheat field dataset (MWWFD) included 1,228 RGB images (500 × 500) and 1,228 corresponding depth images (500 × 500). Broad-leaf and grass weeds in images were labeled using LabelImg; 1,105 images were used for training, and 123 images for testing.

The deep learning framework used is TensorFlow, GPU is NVIDIA RTX2080Ti, CPU is Intel(R) Core(TM) i7-7800x CPU @ 3.50 GHz. The multi-modal weeds detection network was subjected to end-to-end training with backpropagation and stochastic gradient descent methods. For RPN networks, each mini-batch arises from a single image that contains many positive and negative example anchors. Some proposals generated by the region proposal network (RPN) overlapped significantly. To reduce redundancy, we performed non-maximum suppression (NMS) and set the threshold of *IoU* at 0.7. Other training hyperparameters are shown in Table 1. We adopted the weight sharing strategy in the training process, which has been proven effective in many studies because it greatly reduces model complexity and running time (Lecun and Bottou, 1998). Gupta et al. (2016) proved that features learned from depth images are complementary to RGB features even if a CNN based on depth images is supervised and trained by a CNN based on RGB images, and training a network with shared weights is effective.

**TABLE 1:** Training parameters of multi-modal weeds detection network.

Parameters	Value
Initial learning rate	0.001
Momentum	0.9
Weight decay	0.0001
Iteration per epoch	1000
Number of epochs	300

### Evaluation Methods

**FIGURE 4:**
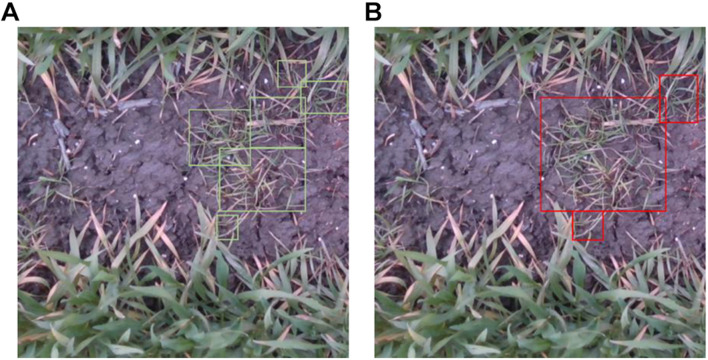
**(A,B)** Different labeling results of the same image.

Mean average precision (*mAP*) is a common and reliable measure of model performance in the detection of multi-category objects.


(9) $$A\,P=\int_{0}^{1}P\,\left(R\right)\,dR$$

where *P* denotes the precision and *R* denotes the recall. *m*AP is APs averaged over all categories. However, in the detection of weeds in wheat fields, labeling was complicated by the cluster growth of weeds. As shown in Figure 4, labeling affected the evaluation of detection precision. Therefore, we used *mAP* to evaluate model detection performance, and intersection over ground truth (*IoG*), which is the quotient of the intersection and union of the detected and labeled datasets, to evaluate the overall precision of weed detection.

## Results

### Evaluation of PHA Image Quality

**FIGURE 5:**
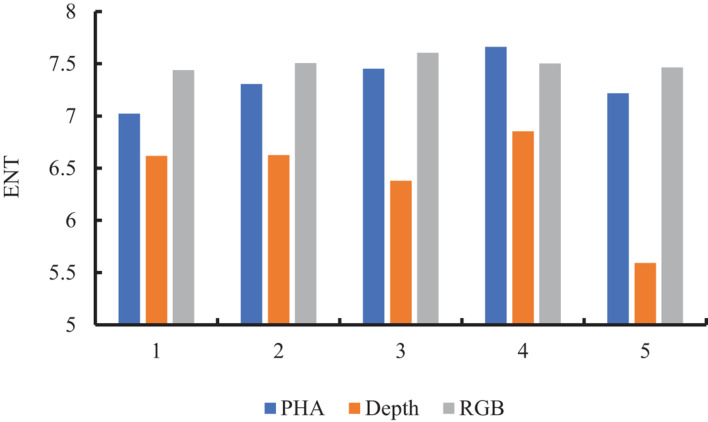
Entropy values of PHA, RGB, and depth images.

**FIGURE 6:**
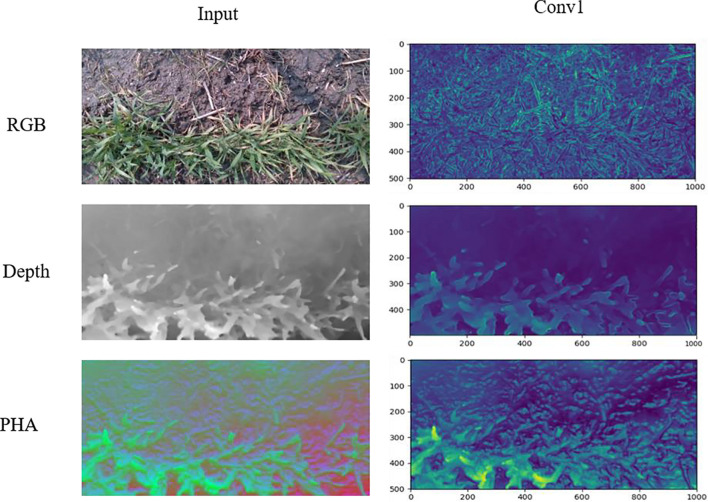
Feature maps from first convolutional layers in VGG16 network of PHA, RGB, and depth images.

We compared the structures of PHA and RGB images from two aspects for suitability in CNN-based feature learning. As shown in Figure 5, the entropy values of PHA and RGB images were closer to each other than to that of depth images, suggesting that they contained more information and were more closely correlated than depth images. Comparison of output from the first convolutional layer of VGG16 showed that PHA images well retained the height information in depth images, with yellow areas in the feature map representing a higher wheat canopy (Figure 6). The depth images had pixels with uniform color in soil and weeds areas, while PHA and RGB images had similar textures, which also indicated their similarity. These comparisons indicated that PHA images obtained by recoding depth images were similar to RGB images in terms of information and structure and were more suitable than depth images for CNN-based feature learning.

### Detection of Weeds in Wheat Fields With Different Datasets

The precision of weeds detection with different datasets is shown in Table 2. Detection based on the PHA dataset was significantly better than on the depth dataset, which confirmed that PHA was more suitable for CNN-based feature learning. Comparison of weeds detection with the three single-modal datasets showed that RGB images were superior in the detection of broad-leaf weeds. Depth and PHA images had similar detection performance regardless of weeds species, and PHA images had the best results in grass weeds detection. The geometric features extracted from PHA images effectively distinguished wheat from the various weeds species.

**TABLE 2:** Weeds detection with single-modal datasets.

Dataset	Backbone	mAP of broad-leaf weeds (%)	mAP of grass weeds (%)	IoG (%)
RGB	VGG16	38.5	24.7	77.6
Depth	VGG16	11.6	11.7	42.1
PHA	VGG16	24.6	25.2	56.9

### Detection With Multi-Modal Datasets in Multichannel Network Architectures

We compared the detection of weeds in wheat fields with different multi-modal datasets and network architectures (Table 3). Dual-VGG16 is the direct superimposition of the last layers of feature maps of different modal images regardless of feature learning in the remaining convolutional layers. Direct superimposition of feature maps reduced precision compared to detection based on single-modal RGB images (Table 2). This is consistent with previous work (Gupta et al., 2016) showing that information mapping in the same scene varies across modalities, and direct fusion can cause the divergence of detection results and diminished precision. Therefore, the complementarity of different modality datasets was considered in network design, and detection performance was optimized by fusing feature maps from different convolutional layers. By comparing the performance of the same model in different datasets, it can be proved that the PHA image obtained by recoding is more conducive to weeds detection. The detection precision (mAP) of grass and broad-leaf weeds with correlated detection net (RPN-Corr) was 29.9 and 39.3%, respectively, and the overall precision (IoG) was 81.4%.

**TABLE 3:** Weed detection with multi-modal datasets in multichannel network.

Dataset	Backbone	mAP of broad-leaf weeds (%)	mAP of grass weeds (%)	IoG (%)
RGB-D	Dual-VGG16	36.2	23.5	72.6
RGB-PHA	Dual-VGG16	37.1	24.2	73.5
RGB-D	RPN-Corr	37.9	25.1	75.2
RGB-PHA	RPN-Corr	39.3	29.9	81.4

### Ensemble Learning Strategy

**FIGURE 7:**
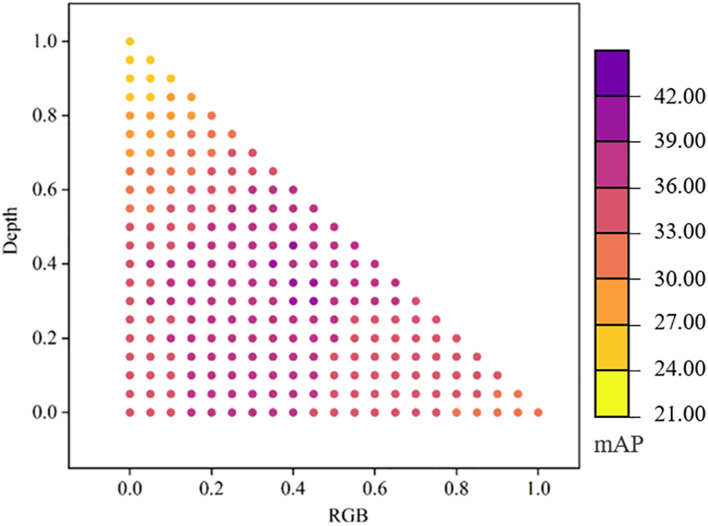
Ensemble learning results of RGB-specific, depth-specific, and RGB-D-correlated models with different weight assignments.

**FIGURE 8:**
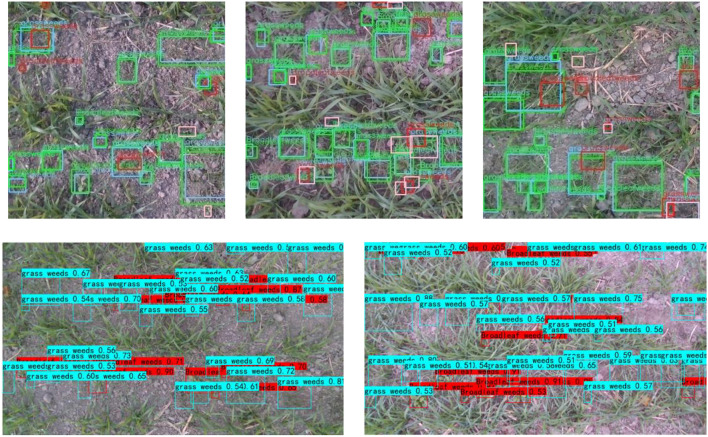
Weeds detection with images of wheat fields. The three graphs in the first row show the detection results of the test set, where green indicates true positive cases, blue indicates corresponding labeling results, pink indicates false negative cases, and red indicates false positive cases. The two graphs in the second row show the detection results of our method on images outside the MWWFD.

While taking into account the complementarity of multi-modal datasets, the independence of datasets was exploited through an ensemble learning strategy. Three independent detection models (RGB-specific, depth-specific, and RGB-D-correlated) were trained, and weights were assigned according to Eq. (3), with results as shown in Figure 7. The detection precision was improved compared to a correlated detection net. When α = 0.4 and β = 0.3, *mAP* = 39.6%. The detection precision of broad-leaf and grass weeds was 42.9% and 36.1%, respectively, and *IoG* = 89.3%. Figure 8 shows weeds results in different images. Notably, we still found some false positive cases on the test set. These cases may be caused by labeling errors. Due to complex field conditions and low image resolution, some fine weeds may be omitted in labeling. The existence of multiple labeling methods in the same weed area is also the main reason for false positive cases. Therefore, we proposed a new accuracy evaluation method, hoping to avoid the influence of such situation on detection results. In the second row of Figure 8, we can find that our method can well realize weed detection in wheat field under natural environment when there is no labeling information. Most areas of both grass and broad-leaf weeds were detected, and the majority of wheat leaves were correctly recognized even with the overlap of leaves in the fields.

## Discussion

### Weeds Detection in Wheat Fields Based on Multi-Modal Information

Previous work in the accurate detection of weeds in wheat fields with information technology mostly used single modalities, such as spectral information and RGB images. However, because of the similar leaf shape and canopy structure of grass weeds and wheat, there are few differences in RGB image features and in reflectance spectra at characteristic wavelengths, which makes the use of modal information difficult for grass weeds detection (Gómez-Casero et al., 2010). We fused depth images with RGB images, extracted geometric features such as height from PHA images, and used multi-modal information for the effective detection of weeds in wheat fields. In the proposed three-channel weeds detection network, feature maps from different convolutional layers were fused using the concept of multiscale object detection. Ensemble learning was carried out at the decision level based on the independence and complementarity of different modalities, which effectively improved weeds detection precision. However, weight assignment in ensemble learning still relied on hand-designed weight gradient experiments, and detection precision remained suboptimal. Weight assignment methods should be further studied.

### Application of Different Machine Learning Algorithms in Weeds Detection

The selection and improvement of machine learning algorithms are a focus in the development of weeds detection technologies. The accuracy and real-time of the detection algorithm determine whether it can be applied in practical agricultural production. In recent years, deep learning methods based on CNN have been widely used in weeds detection with the advantage of end-to-end, avoiding the influence of extraction of manually designed features on detection results (Huang et al., 2018a). This involves calculation of coordinates of bounding boxes around object objects and generates detection results in which the size of the predicted bounding box matches that of the actual weeds object, which improves the precision of weeds detection (Hall et al., 2017). We added a input channel and multi-modal feature fusion blocks, and realized high-precision weeds detection in wheat field through use of multi-modal information effectively. However, compared to traditional machine learning algorithms (Siddiqi et al., 2014; Xu et al., 2020a), the proposed weeds detection algorithm still suffers from a high computational load and low computational efficiency. Although weight sharing was used in training to reduce the computational burden, the demand on the hardware was still high. In future work, we will explore model compression to improve detection efficiency while maintaining precision.

### Weeds Detection in Complex Wheat Field Background

Data (images) of a single category and simple background are usually used in weeds detection, which is limited to the early stage of wheat growth (Nieuwenhuizen et al., 2010; Tellaeche et al., 2011). We considered two growth periods with high incidence of weeds in wheat fields, and the cultivation conditions of field experiments were in line with the actual situation. A weeds detection model based on multiscale object detection was suitable to detect weeds areas of different sizes. However, due to shielding of wheat and weeds leaves in fields, RGB-D images from a single perspective failed to capture the information of the shielded objects. Therefore, we will adopt a multi-perspective approach for image acquisition to mitigate the effect of leaf shielding on weeds detection.

## Conclusion

We proposed a three-channel weeds detection method based on multi-modal information by fusing RGB and depth images and applying the concept of multiscale object detection, which effectively improved the precision of weeds detection in wheat fields. The single-channel depth image is recoded, and the resulting PHA images were more similar in structure to RGB images and more suitable for CNN-based feature learning. The results showed that when the same network was used, weeds detection precision based on PHA images was 1.35-fold of that based on depth images. And the independence and complementarity of the two modalities of RGB and depth images were taken into account, and a three-channel weeds detection network was designed from the perspective of feature- and decision-level fusion. The results showed that the model could effectively detect different species of weeds in wheat fields (*IoG* = 89.3%).

## Data Availability Statement

The original contributions presented in the study are included in the article/supplementary material, further inquiries can be directed to the corresponding author.

## Author Contributions

JN, YZ, XJ, and WC designed the research. JN and KX developed the algorithms. KX, ZJ, and SL performed the research. KX analyzed the data and wrote the manuscript. All authors have read and approved the final manuscript.

## Conflict of Interest

The authors declare that the research was conducted in the absence of any commercial or financial relationships that could be construed as a potential conflict of interest.

## Publisher’s Note

All claims expressed in this article are solely those of the authors and do not necessarily represent those of their affiliated organizations, or those of the publisher, the editors and the reviewers. Any product that may be evaluated in this article, or claim that may be made by its manufacturer, is not guaranteed or endorsed by the publisher.
